# Incubation behavior adjustments, driven by ambient temperature variation, improve synchrony between hatch dates and caterpillar peak in a wild bird population

**DOI:** 10.1002/ece3.3446

**Published:** 2017-10-10

**Authors:** Emily G. Simmonds, Ben C. Sheldon, Tim Coulson, Ella F. Cole

**Affiliations:** ^1^ Department of Zoology University of Oxford Oxford UK

**Keywords:** climate change, great tits (*Parus major*), incubation behavior, mismatch, phenology, phenotypic plasticity

## Abstract

For organisms living in seasonal environments, synchronizing the peak energetic demands of reproduction with peak food availability is a key challenge. Understanding the extent to which animals can adjust behavior to optimize reproductive timing, and the cues they use to do this, is essential for predicting how they will respond to future climate change. In birds, the timing of peak energetic demand is largely determined by the timing of clutch initiation; however, considerable alterations can still occur once egg laying has begun. Here, we use a wild population of great tits (*Parus major*) to quantify individual variation in different aspects of incubation behavior (onset, duration, and daily intensity) and conduct a comprehensive assessment of the causes and consequences of this variation. Using a 54‐year dataset, we demonstrate that timing of hatching relative to peak prey abundance (synchrony) is a better predictor of reproductive success than clutch initiation or clutch completion timing, suggesting adjustments to reproductive timing via incubation are adaptive in this species. Using detailed in‐nest temperature recordings, we found that postlaying, birds improved their synchrony with the food peak primarily by varying the onset of incubation, with duration changes playing a lesser role. We then used a sliding time window approach to explore which spring temperature cues best predict variance in each aspect of incubation behavior. Variation in the onset of incubation correlated with mean temperatures just prior to laying; however, incubation duration could not be explained by any of our temperature variables. Daily incubation intensity varied in response to daily maximum temperatures throughout incubation, suggesting female great tits respond to temperature cues even in late stages of incubation. Our results suggest that multiple aspects of the breeding cycle influence the final timing of peak energetic demand. Such adjustments could compensate, in part, for poor initial timing, which has significant fitness impacts.

## 1 INTRODUCTION

For species living in seasonal environments, reproductive success can be maximized by timing reproduction to coincide with annual peaks in resource abundance (Lack, 1968; Nussey et al., 2005; Parmesan, 2007; Perrins & McCleery, 1989; Van Noordwijk, McCleery, & Perrins, 1995). The timing of these resource peaks can vary considerably between years in response to environmental variation. To achieve synchrony across years, iteroparous animals must exhibit plasticity in their reproductive phenology. If the resource tracked is another species that themselves track environmental conditions, this can be particularly challenging. Synchrony between trophic levels can be achieved and maintained simply by a shared sensitivity to temperature cues; however, interacting species may differ in their sensitivity to environmental variation. Plants and insects respond more directly to temperature changes than homeotherms (e.g., temperate mammals and birds); consequently, the ability of the homeotherm to track the resource species may be constrained (Gaillard et al., 1993; Plard et al., 2014; Van Noordwijk et al., 1995; Visser et al., 1998). Successful matching is dependent on whether animals can perceive ambient temperature and utilize this as a cue to predict optimal reproductive timing in a given year. Such mechanisms, which ensure matching in an interannually varying system, could potentially be disrupted by novel climatic change, leading to mismatches between predators and resources (Gienapp, Hemerik, & Visser, 2005; Gienapp, Reed, & Visser, 2014). For instance, cues which previously predicted the timing of peak prey abundance may no longer do so if climate patterns are altered. Understanding the factors that constrain the extent to which animals can track their resource is important for predicting future levels of mismatch. Determining which elements of the reproductive cycle are flexible and their sensitivity to temperature is a key component of this.

The most commonly studied aspects of reproductive phenology have been those which are easily observable: clutch initiation date (Charmantier et al., 2008; Lack, 1958; Nussey et al., 2005; Schaper et al., 2012; Visser et al., 1998), clutch size (Balen, 1973; Haartman, 1969; Haftorn, 1981; Kluiver, 1950; Lack, 1955, 1958; Perrins, 1965, 1979), birth date (Plard et al., 2014), flowering date (Menzel et al., 2006), and hatch date (Cresswell & McCleery, 2003; Tomas, 2015). However, it is well established that there is also considerable variation in other aspects of the reproductive cycle, such as incubation behavior (Álvarez & Barba, 2014; Ardia, Pérez, & Clotfelter, 2010; Cresswell & McCleery, 2003; García‐Navas & Sanz, 2011; Hepp, Kennamer, & Johnson, 2006; Lord, McCleery, & Cresswell, 2011; Matthysen, Adriaensen, & Dhont, 2010; Stenning, 2008), conception date (Scott, Asher, Archer, & Littlejohn, 2008), and gestation length (Asher et al., 2005; Moyes et al., 2011; Racey & Swift, 1981; Scott et al., 2008). The phenology of many of these reproductive behaviors cannot be observed directly; nonetheless, they could have a significant role in determining the timing of the peak energetic demands of reproduction, usually during offspring rearing. Mammals primarily have one method of flexibility after a reproductive event has been initiated, gestation period. This has been shown to vary in response to food availability and temperature in several species (Asher et al., 2005; Moyes et al., 2011; Racey & Swift, 1981; Scott et al., 2008). In contrast, for bird species, there are several different mechanisms which can alter phenology (hatch date and consequently timing of peak food demand), after a reproductive event has begun and right up until hatching.

The beginning of the reproductive effort for birds is the building of a nest and the onset of egg laying. The lay date of the first egg of a clutch has been well studied and is highly variable (Charmantier et al., 2008; Lack, 1955, 1958; Perrins, 1965, 1979; Van Noordwijk et al., 1995; Visser et al., 1998), with annual shifts of up to several weeks in some species. Changes in lay date have been extensively linked to changes in early spring temperatures (Charmantier et al., 2008; Lack, 1955, 1958; Perrins, 1965, 1979; Schaper et al., 2012; Van Noordwijk et al., 1995; Visser et al., 1998) and represent a plastic response to the environment (Charmantier et al., 2008). Once egg laying has commenced, the majority of birds lay a maximum of one egg per day until their clutch is complete. Therefore, the size of a clutch and the rate of egg laying can delay or advance hatch date. Clutch size and egg laying rate have been shown to vary based on timing of laying, with clutch sizes decreasing as clutch initiation dates become later (Balen, 1973; Haartman, 1969; Haftorn, 1981; Kluiver, 1950; Lack, 1955, 1958; Matthysen et al., 2010; Perrins, 1965, 1979).

Incubation behavior can also impact the timing of hatching in birds species, acting via two main mechanisms. First, the onset of incubation can be advanced or delayed, relative to when a clutch is completed. Second, the duration of the incubation period can be adjusted based on the intensity of incubation effort. Variability in the relative onset of incubation has been demonstrated across a diverse range of bird species (e.g., *Paridae* and *Anatidae*) and can vary by up to a week either side of clutch completion (Álvarez & Barba, 2014; Cresswell & McCleery, 2003; García‐Navas & Sanz, 2011; Hepp, 2004; Loos & Rohwer, 2004; Lord et al., 2011; Matthysen et al., 2010; McClintock, Hepp, & Kennamer, 2014; Stenning, 2008). Such changes are also known to have knock‐on impacts on reproductive success. Beginning incubation prior to completion of a clutch can increase hatching asynchrony and lead to rapid brood reduction in years of poor resources (Álvarez & Barba, 2014; Ardia et al., 2010; García‐Navas & Sanz, 2011; Lord et al., 2011; Stenning, 2008). Variation in incubation onset has been linked to changes in spring temperatures both experimentally (Álvarez & Barba, 2014; Bryan & Bryant, 1999; Vedder, 2012) and through observational studies (Cresswell & McCleery, 2003; Matthysen et al., 2010). Incubation duration and intensity have been less extensively studied but have also been shown to vary with temperatures and potentially with individual condition (Ardia et al., 2010; Conway & Martin, 2000; McClintock et al., 2014).

Although each aspect of incubation behavior has been shown to vary and have some relationship with temperature, the precise temperature cues that trigger variation in these traits are, as yet, unknown. Identifying the temperature metric (mean, maximum, minimum, or temperature range) driving variability in each aspect of incubation behavior, in addition to the temporal window during which these cues are important, is necessary for understanding how incubation behavior could be used to improve hatching synchrony. Alterations to incubation behavior are likely to be most important when temperatures fluctuate throughout the spring (for instance, when initial warming suddenly turns cold). Consequently, it is important to understand the cues used and the limits of plasticity in different elements of incubation behavior in order to accurately predict how hatching timing might change under different climate scenarios. If different aspects of incubation behavior respond to different temperature cues, the different aspects could change at varying rates in the future. In order to determine the role of onset, duration, and intensity of incubation in the final timing of hatching, a detailed assessment of all aspects of incubation behavior is required.

We seek to conduct a detailed study of the incubation behavior of wild great tits (*Parus major*), exploring the extent to which they adjust incubation to improve timing of chick hatching in relation to the peak abundance of their prey species, winter moth caterpillars (*Operophtera brumata*). Passerine songbirds are a good study system to address questions of plasticity and constraints in multiple aspects of the breeding cycle because their reproductive phenology has been extensively studied (Charmantier et al., 2008; Lack, 1955, 1958; Perrins, 1965, 1979; Van Noordwijk et al., 1995; Visser et al., 1998) and, particularly for nest box breeding populations, phenology can be easily monitored. Great tits start incubation gradually, beginning at a few hours each night and increasing to cover the whole night several days prior to clutch completion (Haftorn, 1981). There is then a transition to begin incubating during daylight hours, which again gradually increases up to a point when almost the entire day is spent incubating (Haftorn, 1981). The start of daytime incubation is generally recognized to be the start of true incubation (Haftorn, 1981). Identifying the precise onset of full incubation is necessary to characterize different components of incubation behavior; however, it is challenging to achieve as nest observations alone are insufficient (Stenning, 2008).

Here, we carry out a thorough exploration of the causes and consequences of within‐population variance in the various components of incubation behavior. First, we explore whether great tits improve their reproductive success, and synchrony with their food source, through adjustments to incubation behavior. We then investigate the mechanisms behind these patterns by quantifying the extent to which different aspects of incubation behavior vary, exploring whether this variation can be explained by temperature cues, and ultimately determining which aspects of incubation behavior are important in improving synchrony with the caterpillar peak. We thus address five key questions:

1. Is reproductive success better explained by timing of hatching relative to the caterpillar peak than timing of clutch initiation relative to the caterpillar peak?
2. Do adjustments to timing of hatching made after clutch initiation improve synchrony between chick hatching and the caterpillar peak?
3. How much within‐population variation exists in (a) onset of incubation relative to clutch completion, (b) incubation duration, and (c) incubation intensity?
4. Can this observed variability be explained by ambient temperature cues, and if so, which temperature measures best capture variation?
5. To what extent do these three aspects of incubation behavior (relative onset, duration, and intensity) contribute to improving synchrony between timing of chick hatching and the caterpillar peak?

## 2 METHODOLOGY

### 2.1 Data collection

#### 2.1.1 Long‐term breeding timing

The nest box breeding great tit population of Wytham woods has been studied since 1960 using a standardized procedure (Perrins, 1965; Perrins & McCleery, 1989). Nest boxes (*n* = 1,203 with an average of 225 occupied by great tits each year) are visited weekly from early April. During these weekly checks, nest stage and number of eggs are recorded, and the date that a female initiated her clutch is then inferred by assuming a laying rate of one egg per day and counting back from the number of eggs observed on the weekly check. When at least three eggs are present, they are weighed so that species can be assigned (blue tits, coal tits (*Periparus ater*), and marsh tits (*Poecile palustris*) also use the Wytham nest boxes). Clutch size is defined as the maximum number of eggs observed in the nest. Date of hatching is established by visiting the nest on the estimated hatch date (date of clutch completion plus 11 days) and then every other day until the eggs hatch. If eggs have hatched prior to the hatch check, the largest chicks are weighed and assigned age based on their weight. Parents are identified at the nest either remotely using RFID (radio‐frequency identification) scanners (all previously trapped birds are fitted with RFID tags) or by catching using spring‐loaded nest box traps. All surviving nestlings are tagged with uniquely identifiable metal leg rings and RFID tags at 2 weeks of age. Nests are then checked postfledging to determine the number of chicks that left the nest.

The timing of caterpillar peak abundance is taken to be the median date on which caterpillars descend to the ground to pupate. These data have been collected as part of a long‐term study in Wytham woods, supplied by Dr L. Cole.

#### 2.1.2 In‐nest temperature data collection and incubation onset identification

During the 2014 breeding season, in‐nest temperature was recorded at 163 great tit nests using iButton thermometers (DS1921G‐F5, accurate to ±1°C; HomeChip Ltd) set to record temperature every 20 min. These iButtons were secured in the nest cup by wrapping blunted garden wire around the iButton and using the protruding ends to anchor the iButton in the nest material. The iButtons and wire were then sealed into small cotton pouches. Pouch color was matched as closely as possible to the nesting material to minimize visibility to the female great tit (for further details, see Figure 1 in the supporting information, S1). iButtons were placed in every second great tit nest discovered across the woodland, throughout the season, ensuring a spatially and temporally even spread of sampling (163 of the 337 great tit nests). iButtons were placed in nests prior to the start of incubation (eggs were cold to the touch). Incubation in great tits occurs gradually (Haftorn, 1981), and therefore, eggs can feel cold even after daytime incubation has begun if a fieldworker visits when the female is not incubating. Consequently, only nests which showed, from in‐nest temperatures, at least 1 day of nonincubation after placement of the iButton were included in our analyses. Four nests that did not meet this criteria were removed from the analysis.

**Figure 1:**
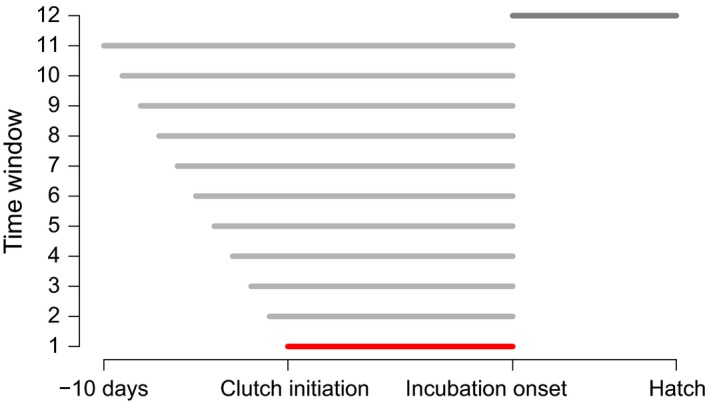
Plot of 12 sliding windows of temperature. Time through the breeding cycle is shown on the *x*‐axis and name of the window on the *y*‐axis. Window 12, dark grey, was used only for analyses of incubation duration and covers the incubation period. The minimum window, from clutch initiation date to the onset of incubation, is highlighted in red

Of the 163 iButtons placed, 109 were retrieved (54 disappeared from the nest and were assumed to have been removed by the resident great tit). There was potential for bias to be created if females who removed iButtons had a tendency for a certain incubation behavior. However, there was no statistical difference between the clutch completion to hatch period for those females who removed iButtons and those that did not (full analysis reported in supporting information S3). Of the retrieved iButtons, a further six nests were removed from analyses because they were abandoned, three prior to incubation onset and three after incubation but prior to hatching. There was no significant association between abandonment and whether a nest had an iButton or not (χ^2^ = 3.06, *df* = 1, *p* = .08, *N* = 337). A further six iButtons were removed from analyses due to indistinct readings, probably due to deep burial in the nest by the resident female. We therefore present data from the remaining 93 nests where onset of daytime incubation was clearly identifiable.

Date of onset of daytime incubation was determined by combining in‐nest temperature measures with hourly local ambient temperature measures (see below for details of ambient temperature data collection) and calculating the difference between the two. Every in‐nest temperature was paired with a local ambient reading from the same hour. Each in‐nest iButton was matched to the closest ambient temperature iButton using GPS coordinates in ArcGIS (Environmental Systems Research Institute (ESRI), 2011). In order to identify the onset of daytime incubation, it was necessary to distinguish when a female was incubating in the daytime. This posed a methodological challenge because the temperature readings from the iButtons did not represent the exact egg temperature due to different conductive properties of the egg and the iButton. iButtons were also prone to slight burial and movement within the nest cup based on female behavior, potentially altering the temperature readings. As a result, it was necessary to calibrate each iButton daily. The in‐nest iButton was calibrated to its daily position by selecting a period each day, when the female is known to be incubating (the period just after dark, c. 7 p.m., to midnight (Haftorn, 1981)), and using the temperatures recorded at this point as a threshold for what can be considered an incubating temperature. In‐nest temperature was calculated for this every night and temperatures over 4°C greater than ambient were taken to indicate incubation. This cutoff was chosen in order to avoid classing small deviations (1 or 2°C) caused by ambient temperature differences, as incubation. All in‐nest iButtons showed differences of <1°C from local ambient temperature during the active day (7 a.m. to 7 p.m.) prior to the onset of daytime incubation; therefore, we are confident a 4°C cutoff is sufficient to indicate incubation. The minimum of these incubating temperatures was then taken as the minimum incubation temperature for the focal nest and current iButton position. Recorded temperatures during the following active day (7 a.m. to 7 p.m.), which exceeded the defined threshold for a given nest and day, were classed as showing incubation is taking place. The onset of daytime incubation was consequently defined as the day when at least 50% of the active‐day recordings were classed as “incubating” (see Figure 2 in the supporting information, S2 for further details of how thresholds were defined).

**Figure 2:**
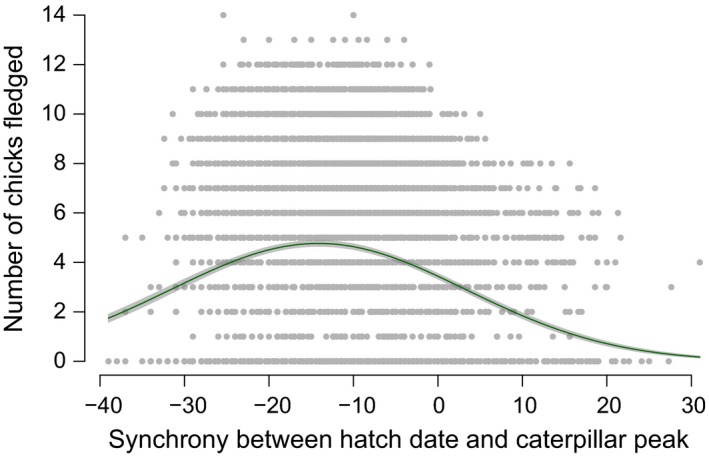
The number of chicks fledged against hatching synchrony. Data is from 1960 to 2014. The plotted line is generated from a Poisson GLM with fixed effects of clutch initiation date relative to caterpillar timing, its quadratic, clutch size, hatch date relative to caterpillar timing, its quadratic, and section of the woodland. The shaded area represents the 95% confidence interval of the plotted line

#### 2.1.3 Ambient temperature data collection

Local ambient temperature was collected via a grid of ambient temperature iButtons (DS1923‐F5, accurate to ±0.5°C; HomeChip Ltd) set to measure absolute temperature every 30 min. A total of 200 of these ambient temperature iButtons were distributed in a grid system across Wytham woods with positions chosen to reflect the density of nest boxes. For further details, see (Cole & Sheldon, 2017).

### 2.2 Statistical analyses

#### 2.2.1 Is reproductive success better explained by timing of hatching relative to the caterpillar peak than timing of clutch initiation relative to the caterpillar peak?

Whether hatch timing relative to winter moth caterpillar peak abundance (taken as the median date on which caterpillars were observed descending to pupate) is important for reproductive success was tested using a Poisson generalized linear model (GLM) with number of fledglings as the response variable. Fixed effects were hatching synchrony (observed hatch date minus median caterpillar date of that year) and its quadratic. Clutch size, clutch initiation synchrony (observed clutch initiation date minus median caterpillar date of that year), its quadratic, and section of the woodland were also included as explanatory variables to take account of number of eggs laid, individual condition, and local habitat quality.

#### 2.2.2 Do adjustments to timing of hatching made after clutch initiation improve synchrony between chick hatching and the caterpillar peak?

In order to address the overarching question of whether adjustments after clutch initiation improve synchrony between chick hatching and the caterpillar peak, we address two subquestions.

1. *Is annual population‐level variance in hatching timing reduced through incubation adjustments?* The annual variance of hatch dates and clutch initiation dates was calculated from 1960 to 2014. To take account of the potential influence of changes in clutch size, we also calculated the variance of clutch completion dates. The variances of clutch initiation date, clutch completion date, and observed hatch date were compared using an ANOVA including a fixed effect of year.
2. *Is synchrony with the food source improved by adjustments made between clutch initiation and hatching?* To distinguish whether the observed synchrony between hatch dates and caterpillar peak abundance was a significant improvement to the null expectation of this synchrony, without incubation alterations, a paired *t* test was conducted. Observed synchrony was calculated as the difference between each nest's hatch date and the woodland annual caterpillar timing. All synchrony values had 13 added to them as 13 days prior to the caterpillar peak was indicated to be the optimal timing of hatching (see above for the analysis of reproductive success). This gives an index with negative values indicating hatch timing earlier than optimum, positive values indicating hatch dates later, and 0 indicating optimal synchrony. The null expectation assumed no incubation behavior alterations and was calculated as the clutch completion date plus 14 days (to represent duration of incubation) minus annual caterpillar timing. Synchrony index measures were then squared in order to remove negative values.

#### 2.2.3 How much within‐population variation exists in (a) onset of incubation relative to clutch completion, (b) incubation duration, and (c) incubation intensity?

Using the identified onset of full incubation for the 2014 breeding season, we calculated two primary aspects of incubation behavior: the relative onset (interval between clutch completion and start of daytime incubation) and duration (interval between onset of incubation and observed hatch date). In addition, we also quantified the daily intensity of incubation effort. Incubation effort for each day was determined by calculating the number of 20‐min periods during the active day (7 a.m. to 7 p.m.—the active day) that exceeded the threshold temperature for incubation, as a proportion of the total number of active‐day recordings. The range, mean, and variance of each of these aspects of incubation behavior were calculated.

Relationships between each of the aspects of incubation behavior were also tested. Linear models (LMs) and GLMs were run for each combination of behaviors. Due to sequential timing, relative incubation onset could not be causally influenced by either intensity or duration. However, the onset itself could influence the intensity of incubation effort and consequently impact the duration of daytime incubation. Furthermore, other factors, such as individual condition and clutch size, could also impact incubation effort. These associations were tested in two analyses. The first was a binomial GLM, with mean intensity of incubation as a response variable and relative incubation onset as an explanatory variable, accounting for clutch size and clutch initiation date (a proxy for individual condition (Rowe, Ludwig, & Schluter, 1994)). The second was a LM with incubation duration as the response variable and mean intensity, relative incubation onset, and clutch size as explanatory variables. The duration of daytime incubation should be a result of the amount of incubation required for an embryo to develop and hatch, scaled by the intensity at which it was incubated. As a result, we would expect incubation duration to show a relationship with mean incubation intensity. However, this relationship could also be modulated by other processes, which influence the amount of incubation required. This could be altered by clutch size (Haftorn, 1981) and the amount of prior incubation, through relative incubation onset. A nest that began incubation prior to clutch completion could require a greater intensity of incubation effort for the same duration as a nest that began incubation after clutch completion. This occurs due to accumulated hours of incubation during nocturnal incubation reducing the amount required from full incubation for nests which delay onset.

#### 2.2.4 Can this observed variability be explained by ambient temperature cues, and if so, which temperature measures best capture variation?

For both relative incubation onset and incubation duration, exploratory analyses were conducted using sliding time window methods in order to identify the temporal temperature window and temperature measure, which best explain variance in different components of incubation behavior. This method does not establish a causal link between the behavior of interest and temperature, but instead attempts to identify the time window in which temperature may be most important for determining the behavior in question.

Twelve different length windows were tested for these analyses (Figure 1). The minimum time window (shown in red) was unique to each nest and spanned from the lay date to the date of onset of full incubation, hereafter termed the “laying period.” The time windows then increase in 1‐day increments spanning backward from the start of laying, up to the maximum window of 10 days prior to laying, up until incubation onset (see Figure 1). Window 12 is only used in the analysis of incubation duration and spans from the onset of incubation up until hatching.

Candidate models for model selection were LMs and took the form of two configurations of null model (models with no temperature variables included), which were compared with four configurations of temperature model, each containing a single temperature variable (see Table 1a). Four different temperature measures were tested: mean temperature, mean daily minimum temperature (MMin), mean daily maximum temperature (MMax), and mean daily temperature range (Trange). In total, this gave 44 different explanatory temperature variables (four measures across 11 windows). A total of 176 candidate temperature models were compared with relative incubation onset as the response variable, consisting of 44 different temperature variables in each of the four model configurations and the two null models. For models with incubation duration as a response variable, each candidate model also included a fixed effect of relative incubation onset. As incubation occurs at night prior to the onset of full incubation, any delay or advance in full incubation onset will alter the number of accumulated “incubation hours” and therefore will likely influence the final duration of full incubation. An additional four candidate models were also run due to the additional time window for this variable, totaling 180 candidate models.

**Table 1:** Candidate model configurations for the analysis of incubation behavior. (a) Candidate models for the analysis of relative onset of incubation and incubation duration. Models for incubation duration also include the relative onset of incubation as a fixed variable. (b) Candidate models for the analysis of daily incubation intensity

Model name	Explanatory fixed variables
(a)	
Null 1	Clutch initiation date
Null 2	Clutch initiation date + Clutch size
Temperature 1	Temperature variable
Temperature 2	Temperature variable + Clutch initiation date
Temperature 3	Temperature variable × Clutch initiation date
Temperature 4	Temperature variable + Clutch size
(b)	
Null 1	Clutch initiation date
Temperature 1	Incubation day + Temperature variable
Temperature 2	Incubation day + Temperature variable + Clutch initiation date
Temperature 3	Incubation day + Temperature variable × Clutch initiation date
Temperature 4	Incubation day + Temperature variable + Clutch size

For incubation intensity, binomial generalized linear mixed‐effects models (GLMMs) were used with daily incubation effort as the response variable and temperature and stage of incubation (incubation day) as fixed effects. The random effect of individual was also included to take account of individual differences in incubation effort. Incubation days ran from the onset of full incubation (day 1) to the day prior to hatch day. Hatch date itself was excluded as this is when iButtons were removed. Candidate models each included a single temperature variable from daily mean temperature, daily minimum temperature, daily maximum temperature, and daily temperature range. Five different configurations of model were trialed (see Table 1b). Therefore, a total of 20 candidate models were trialed.

Candidate models were compared using the ΔAIC. The preferred model was defined as the model with the lowest AIC. Models are considered as not significantly different to the preferred model if the ΔAIC is less than 2. It should be noted that as each model including a temperature variable contains at least a partially overlapping or correlated variable (as all temperature measures and windows are likely to be correlated), we would not expect these analyses to always produce any single clearly preferred model.

#### 2.2.5 To what extent do these three aspects of incubation behavior (relative onset, duration, and intensity) contribute to improving synchrony between timing of chick hatching and the caterpillar peak?

The influence of relative incubation onset and incubation duration on the observed synchrony was tested by comparing the observed hatch date synchrony (hatch date minus caterpillar peak timing) to a null expectation of hatch synchrony, if either aspect of incubation behavior had not varied. This is similar to the technique applied to the long‐term data; however, in this instance we are able to distinguish between different aspects of incubation behavior and quantify their impacts independently. A null expectation was created for both relative incubation onset (calculated as the clutch completion date plus the duration of full incubation minus caterpillar timing) and incubation duration (calculated as the clutch completion date plus the relative incubation onset and the mean duration of full incubation in 2014—12 days minus caterpillar timing). The variance of the observed hatching synchrony was compared to the two null estimations using pairwise *F* tests for equality of variance. As there was no logical null estimate for the duration of daytime incubation, isolation of the influence of this element of incubation behavior on mean synchrony was not possible. Therefore, only the influence of relative incubation onset on mean synchrony was tested. This was conducted using a paired *t* test between the observed synchrony and a null estimate with no onset changes.

## 3 RESULTS

### 3.1 Hatching timing relative to the food peak abundance influences reproductive success and is a better predictor than relative clutch initiation timing

The number of chicks fledged showed a significant relationship with observed hatching synchrony (difference between hatch date and caterpillar peak date) (EST = −0.074, *SE* = 0.0037, *p* < .01) and its quadratic (EST = −0.0017, *SE* = 0.00013, *p* < .01) (see Figure 2). Those hatching too early (more than 13 days prior to the caterpillar peak) or too late (less than 13 days prior to the caterpillar peak) fledged fewer young. The number of chicks fledged also showed a significant positive relationship with clutch initiation synchrony, but not the quadratic (EST = −0.00012, *SE* = 0.000099, *p* = .24). Earlier layers fledged more young; however, the effect size was almost half that of hatch timing (EST = 0.036, *SE* = 0.0069, *p* < .01).

### 3.2 Incubation alterations lead to hatch dates having lower variance than clutch initiation or completion dates

From 1960 to 2014, hatch dates in the Wytham woods great tit population showed significantly lower annual variance than clutch initiation dates. Hatch dates had an average variance of 3.7 days less than clutch initiation dates (variance difference = −13.6, *SE* = 0.97, *p* < .01). This difference was partly driven by changes in clutch size, but not exclusively. Variance in clutch completion date was significantly lower than in clutch initiation date (variance difference = 5.9, *SE* = 0.97, *p* < .01) and significantly greater than observed hatch date variance (variance difference = 7.6, *SE* = 0.97, *p* < .001).

### 3.3 Incubation alterations improve synchrony between hatch dates and the caterpillar food peak

Results of a paired *t* test show that the asynchrony between observed hatch dates and caterpillar timing is 3.35 days lower (closer to optimal synchrony) than for the null estimate of hatching asynchrony, if no incubation alterations occurred. This is statistically significant (*T* = −26.6, *df* = 11,438, *p* < .01).

### 3.4 Relative incubation onset, incubation duration, and intensity are highly variable

For the 2014 breeding season, relative incubation onset ranged from 3 days prior to clutch completion up to 12 days after, with an average of four and a half days delay. Incubation duration ranged from 7 to 19 days with an average duration of 11.5 days. Incubation intensity, once full incubation had begun, ranged from 5% to 100% of the active day, with a mean incubation effort of 70%.

The maximum alteration to hatch date, assuming independence of different incubation behaviors and an expected incubation duration of 11.5 days, is an advance of 7.5 days or a delay of 19.5 days.

The mean intensity of incubation showed no significant relationship with either clutch size (EST = 0.04, *SE* = 0.03, *p* = .14) or clutch initiation date (EST = 0.01, *SE* = 0.01, *p* = .42), but did show a significant negative relationship with relative incubation onset (EST = −0.04, *SE* = 0.01, *p* < .001). Incubation duration also showed a significant negative relationship with the relative onset of incubation (EST = −0.45, *SE* = 0.05, *p* < .001), but did not show any significant relationship with either clutch size (EST = 0.03, *SE* = 0.12, *p* = .79) or mean intensity (EST = −3.92, *SE* = 2.09, *p* = .06).

### 3.5 Relative incubation onset varies in response to mean local temperatures around the laying period

Ten candidate models had ΔAIC values of within two of the lowest AIC model, giving 11 candidate models with equal support.

All of these models included mean temperature or mean maximum temperature. The majority (nine of 11) included an additive effect of clutch initiation date or an interaction between clutch initiation date and temperature. Effect sizes for the relationship between temperature and relative incubation onset vary from −5.4 for mean temperature to −0.7 for mean maximum temperature. As all of these models are similar in composition, only the model with the lowest AIC is discussed further (but a full list of model parameters is given in supporting information S4). The model with the lowest AIC included explanatory variables of mean temperature for time window 8, clutch initiation date, and an interaction between the two.

**Figure 3:**
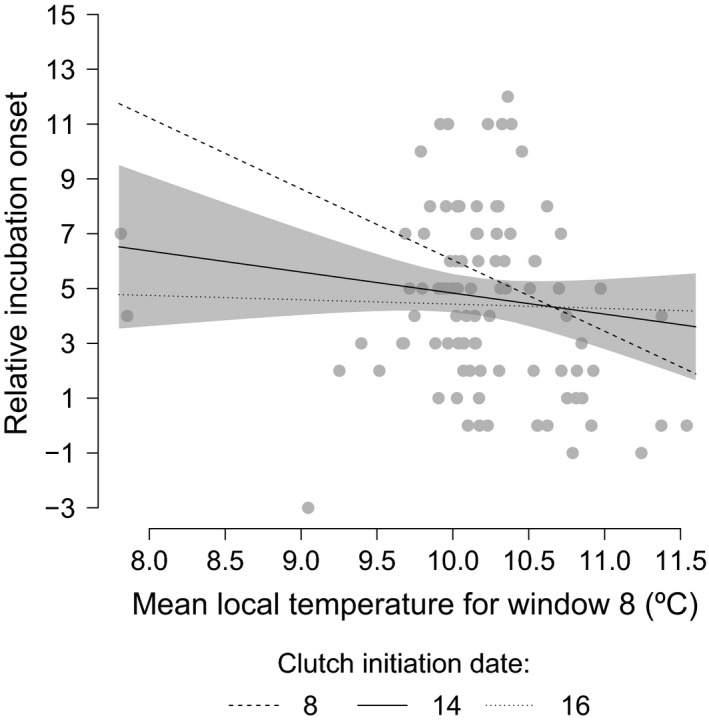
Relative onset of incubation against mean temperature for window 8. The plotted:lines are generated from predictions from linear models. Clutch:initiation dates are held at the 1st quartile value, the median value, and the 3rd quartile value, to illustrate the significant interaction between clutch initiation date and temperature. Shaded area represents the 95% confidence interval for predictions with median clutch initiation date only, to improve the readability

The relative incubation onset had a significant negative correlation with mean temperature for window 8, with individuals starting incubation earlier when mean temperature was higher (5 days advance in the onset of incubation per 1°C increase in mean temperature; Figure 3; EST = −5.03, *SE* = 1.72, *p* = .004). The interaction between clutch initiation date and temperature took the form of early layers having the strongest negative relationship between temperature and their incubation onset and later layers showing almost no relationship with temperature, as illustrated in Figure 3 (EST = 0.3, *SE* = 0.13, *p* = .02). Figure 3 show the relationship including outliers; the relationship was also tested without these data points (mean temperature values <8.5°C). Parameter values did not change considerably on removal of the outliers; consequently, we opted to retain all data. Full output of both analyses is given in supporting information S4.

### 3.6 Variability in incubation duration shows no significant relationship with temperature

The duration of full incubation showed no significant relationship with any temperature measure or temporal window once the relative onset of incubation was taken into account. The candidate model with the lowest AIC included the temperature range for window 11 and the relative incubation onset; however, the effect of temperature was not statistically significant at *p* = .05 (EST = 0.26, *SE* = 0.15, *p* = .09).

Full model selection results and model parameters are given in supporting information S5.

### 3.7 Daily intensity of incubation effort increases with higher daily maximum temperatures

**Figure 4:**
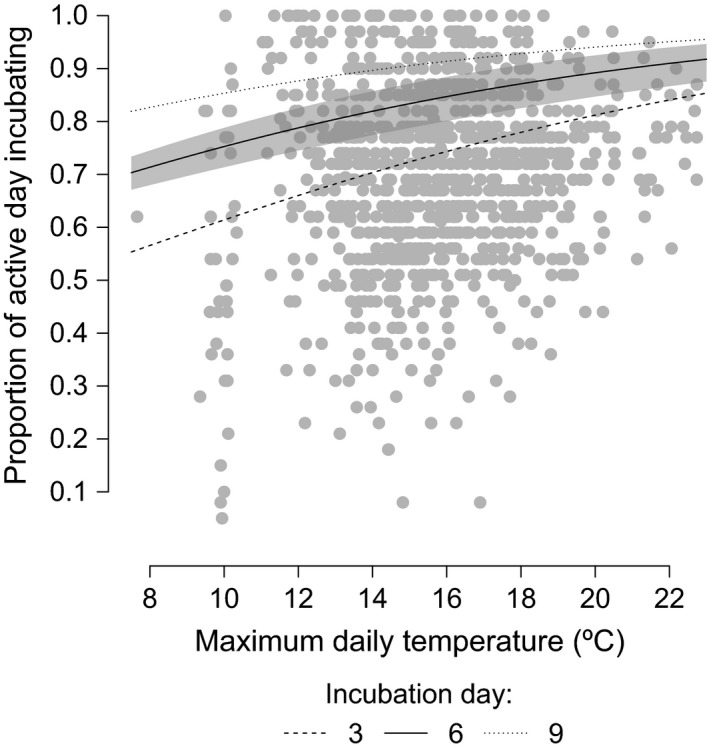
The proportion of the active day spent incubating against maximum daily temperature. The plotted lines are generated from predictions from a binomial GLMM using mean incubation day and one standard deviation above and below the mean incubation day (start of full incubation = incubation day 1). For the purposes of prediction, random effects were ignored. Shaded area represents the 95% confidence interval for the relationship between temperature and intensity when incubation day is 6 only, to improve readability

The proportion of the active day spent incubating was significantly positively correlated with time through incubation (~5% increase per day of incubation) and local maximum daily temperature (~2% increase per 1°C), according to the lowest AIC model. There was also a weak but significant negative interaction between temperature and day of incubation (EST = −0.008, *SE* = 0.001, *p* < .001); with the relationship between temperature and intensity being weaker the further through incubation a bird is. All other candidate model configurations had ΔAIC values of >2. These results show that the further through incubation a female is, and the warmer the daily temperature, the higher the proportion of the day is spent incubating (see Figure 4).

Full model selection results and model parameters are given in supporting information S6.

### 3.8 Changes to relative incubation onset alter the mean, but not the variance, of synchrony between hatch dates and food peak abundance

Null estimations of hatching synchrony, assuming no changes in relative incubation onset or no changes to incubation duration, and the observed hatching synchrony were compared. This allowed us to tease apart the contributions of different aspects of incubation behavior to the observed hatching synchrony for the 2014 breeding season. The inclusion of alterations to incubation duration altered the variance of synchrony among nests, from 44.8 without duration changes to 37.8 for observed hatching synchrony. The inclusion of changes to the relative onset of incubation also showed a difference in variance, but to a much lower extent (1.1 higher than observed variance). None of these differences in variance were statistically significant. *F* test of variances for a baseline with no duration changes compared to observed hatching synchrony showed a ratio of variances of 1.18 (*df* = 92/92, *p* = .21). The *F* test for no onset changes compared to observed hatching synchrony showed a ratio of variances of 1.02 (*df* = 92/92, *p* = .44).

A paired *t* test testing the difference in mean synchrony with and without incubation onset changes showed a significant difference. Mean synchrony was 4.5 days lower, closer to optimum, for observed compared to null synchrony (difference = −4.5, *T* = 13.29, *df* = 92, *p* < .01).

## 4 DISCUSSION

This study examined whether incubation behavior is used to improve the timing of hatching relative to peak resource abundance (winter moth caterpillars), and which temperature cues best predict within‐year variation in incubation. We quantified variability in different aspects of incubation behavior for a single breeding season (the onset relative to clutch completion, duration, and intensity) and explored how such changes are linked to temperature and how they can influence hatch timing. Using a population of wild great tits, we show that across 54 years, individual hatching dates show lower within‐year variance than clutch initiation dates, suggesting that alterations are occurring post‐clutch initiation that are bringing hatching dates closer to the mean. These alterations were found to be driven by both clutch size changes and incubation behavior. Synchrony between hatch dates and caterpillar peak abundance was shown to be significantly improved for observed hatching compared to a null estimation (where incubation behavior was assumed to be the same for all individuals). This suggests that incubation behavior is being used to improve synchrony with peak food availability.

In this study, we were interested in how different aspects of incubation behavior drive the observed post‐clutch initiation adjustments and to identify the temperature cues which best explain this variability. In order to distinguish different elements of incubation behavior, we used a passive method of in‐nest temperature recording. Through this, we were able to accurately distinguish the onset of full incubation and consequently quantify the relative onset, duration, and intensity of incubation. We demonstrate that incubation adjustments are driven by variability in multiple aspects of incubation behavior. The onset of incubation relative to clutch completion, the duration of full incubation, and intensity of incubation effort are all highly variable. Cumulatively, alteration to these behaviors could lead to almost 8 days advance or 20 days delay in hatch timing. At least some of the variation observed in these behaviors can be explained by temperature changes from just prior to the laying period right up until hatching. An interplay between the energetic costs of incubation (Visser & Lessels, 2001) and the fitness costs of timing hatching poorly in relation to peak food abundance could also play a role in determining optimal incubation behavior. However, this study focuses on the role incubation behavior plays in the timing of hatching.

Different aspects of incubation behavior correlated with different temperature variables. Sliding time window analyses identified a critical temporal window of mean temperatures from 7 days prior to laying up until incubation onset (see Figure 3) as the best predictor of variability in relative incubation onset. Warmer mean temperatures during this period corresponded to earlier relative onsets with an advance of approximately 5 days per °C but mediated by an interaction with clutch initiation date. Earlier layers displayed the strongest negative temperature—onset relationship, supporting previous findings that early layers are most able to alter their timing (Cresswell & McCleery, 2003). This suggests that temperatures around laying do act as a cue for the onset of incubation, supporting several previous findings (Álvarez & Barba, 2014; Cresswell & McCleery, 2003; Stenning, 2008). However, the cues for incubation behavior were not found to be uniform across different aspects. While some of the findings from this study support previous work suggesting that mean temperature is an important phenological cue (Álvarez & Barba, 2014; Cresswell & McCleery, 2003; García‐Navas & Sanz, 2011), we also show that for some aspects of incubation behavior, temperature extremes are more important. This demonstrates a need to test multiple temperature measures when considering phenological cues. It should also be noted that temperatures do autocorrelate throughout the year, and between different measures (mean, maximum, minimum, and range), this can make distinguishing a definitive temperature cue difficult.

The different aspects of incubation behavior studied here were not completely independent. Both the intensity and duration of incubation appeared to be partly constrained by the onset of incubation. The daily intensity of incubation effort showed a significant positive relationship with daily maximum temperatures during the incubation period, even when accounting for stage of incubation. In contrast, incubation duration showed no significant relationship with any temporal window or temperature measure tested here. This could arise because incubation duration is constrained due to an interaction between the number of hours of incubation required for development and the relative onset of incubation. Consequently, it may not exhibit plasticity in response to temperature variation, despite changes to intensity. Incubation durations are highly variable, but a significant portion of this variability can be explained by the relative onset of incubation. Mean incubation intensity showed no relationship with clutch size or lay date (proxy for individual condition) but a significant negative relationship with the relative incubation onset. Higher mean intensities corresponded with earlier incubation onsets. This could indicate an attempt by females to advance their hatch date via both onset and duration of incubation. However, this relationship is not shown for duration itself. Incubation duration showed no significant relationship with either clutch size or mean intensity of incubation. It did, however, show a significant negative relationship with relative incubation onset; earlier onsets corresponded to longer durations. The lack of a relationship between intensity of incubation effort and the duration of incubation could be explained by the role of nighttime incubation. As great tits incubate at night from several days prior to clutch completion (Haftorn, 1981), those individuals initiating full incubation prior to clutch completion will have accumulated fewer active incubation hours than an individual who initiates after the clutch is completed. Consequently, females incubating early will need to input more hours of full incubation than those who delayed. This would lead to a modulation of any intensity–duration relationship with females with earlier onset requiring a greater intensity of incubation in order to achieve the same total duration as a female with later onset.

Here, multiple aspects of the breeding cycle have been shown to play a role in achieving synchrony between hatching timing and caterpillar peak abundance. We have demonstrated that elements of incubation behavior alter hatch dates in different ways. The relative onset of incubation significantly alters the mean hatching synchrony to create better matching with the caterpillar peak abundance. The duration of incubation altered the variance of hatching timing, although not significantly in the focal year. The relative onset of incubation had no influence on the variance of hatching timing; therefore, all variance changes are likely attributable to alterations of incubation duration. Although not statistically significant in 2014, we did find that incubation behavior significantly altered variance in hatching timing across long‐term data, which could be driven by duration changes. As a result, incubation duration changes do appear to play a notable role in eventual synchrony between hatching and caterpillar peak abundance across years.

Hatching timing, relative to the caterpillar peak, has a significant impact on reproductive success, and consequently, all alterations discussed here play a key role in fitness. Further to direct effects on the relative hatching timing, incubation alterations could also have other knock‐on fitness impacts. The shifting of incubation onset earlier could influence reproductive success through increases in asynchrony in the hatching of individual eggs within a clutch (Cresswell & McCleery, 2003; Johnson et al., 2013; Lord et al., 2011; Stenning, 2008). This can drive rapid brood reduction in years with little food or when matching is poor, therefore reducing recruitment and having impacts on population dynamics.

When assessing plasticity in different elements of the breeding cycle, it is important to consider the limits and constraints on this flexibility. There is an inherent asymmetry in adjustments to incubation behavior. As demonstrated with females in our study, it is much easier to delay incubation onset than to advance. An advance in incubation onset is bounded by initial lay date, but eggs remain viable many weeks after being laid (Perrins, 1970), so females have more flexibility to delay incubation than they do to advance it (Van Noordwijk et al., 1995). Therefore, if temperatures after laying advance faster than prior to laying, as is occurring for great tit populations in the Netherlands (Visser et al., 1998, 2003), plasticity in incubation behavior alone may not be sufficient to compensate for a late lay date in comparison with subsequently early caterpillar peak. This is demonstrated by reduced reproductive success for this population in years when temperatures change drastically after laying (Van Noordwijk et al., 1995). Additionally, females may be energetically constrained from advancing incubation very early in the spring when resources are scarce (Van Noordwijk et al., 1995).

The patterns shown here are not unique to great tits. Variation in timing after reproduction has been initiated has been shown in blue tits (García‐Navas & Sanz, 2011; Matthysen et al., 2010; Stenning, 2008), tree swallows (Ardia et al., 2010), ducks (Hepp et al., 2006), cervids (Asher, 2007; Asher et al., 2005; Moyes et al., 2011), and bats (Racey & Swift, 1981). In order to predict how populations of temperate species will be impacted by further climate changes, it is essential to consider the mechanisms behind phenological synchrony, the responsiveness to temperature, and their constraints. Many species appear to display considerable flexibility in multiple aspects of their breeding phenology. Therefore, they might have greater potential to adapt to further climatic variability than would be assumed from looking at only single aspects of reproduction. In this study, we demonstrated that great tits do alter incubation behavior in response to ambient temperatures from prior to laying right up until hatching, even adjusting to daily maximum temperature changes during incubation. These adjustments improve the synchrony between hatch timing and caterpillar peak abundance and have knock‐on impacts to reproductive success through this improved matching.

## CONFLICT OF INTEREST

None declared.

## AUTHOR CONTRIBUTIONS

Emily G. Simmonds has been the lead on the conception; design; acquisition, analysis, and interpretation of data; and writing of the manuscript. Dr Ella F. Cole has provided substantial contributions and support to all elements of this manuscript. Professors Ben C. Sheldon and Tim Coulson have provided considerable contributions to the conception and design of this study in addition to interpretation of data and drafting of the work. All authors agree to be accountable for all aspects of this work.

## DATA ACCESSIBILITY

Datasets used for the analyses in this manuscript can be found in the supporting information.
