# Supplementary material for: Incubation behavior adjustments, driven by ambient temperature variation, improve synchrony between hatch dates and caterpillar peak in a wild bird population
Source: Ecol Evol. 2017 Oct 10;7(22):9415–25. doi: 10.1002/ece3.3446 (PMC5696398; doi:10.1002/ece3.3446)
Supplement: Supplementary file 1 [file ECE3-7-9415-s001.pdf]

Supporting Information:

Incubation behaviour adjustments driven by ambient temperature variation improve synchrony between hatch dates and caterpillar peak in a wild bird population

Emily G. Simmonds, Ben C. Sheldon, Tim Coulson and Ella F. Cole

S1: iButton construction and placement

Illustrative photographs of how iButton thermometers were placed in great tit nests.

Figure 1: Photographs of the construction of in-nest iButtons. From left to right: the iButton alone, the iButton with its garden wire anchor, the iButton secured in cloth pouch, the finished iButton placed in a nest amongst the eggs

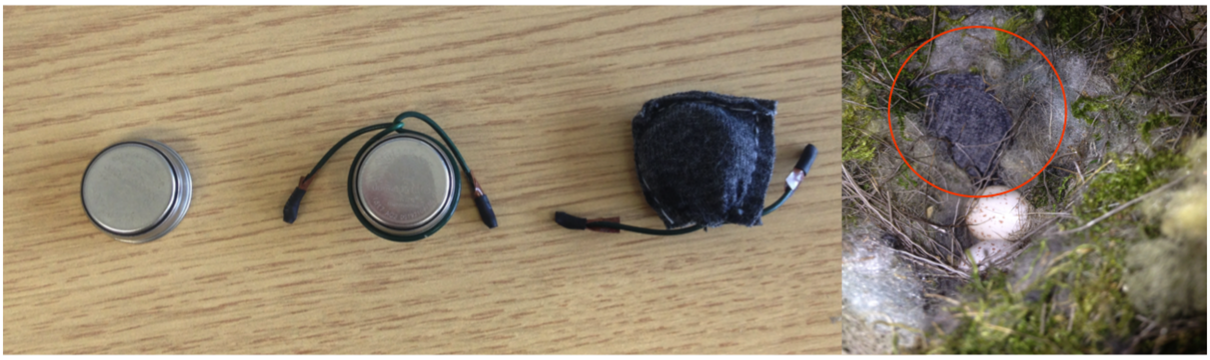

S2: Defining the onset of daytime incubation

The definition of the onset of incubation can be a challenging subject. As incubation is a gradual processes, usually beginning at night and then gradually extending into the active day (daylight hours) creating a clear cut off for when this incubation began is somewhat arbitrary. Haftorn (1981) discusses this dilemma in some length and defines two definitions of incubation onset; onset 'senso stricto', which would refer to the onset of the first incubation (usually at night) and onset of steady incubation (what we term full incubation) when a female's attentiveness reaches optimum. It was the onset of full incubation that we are interested in here and Haftorn (1981) defined this as when 60-85% of the active day was spent incubating (however this was specific to Norway). In a paper by Cresswell and McCleery (2003) on great tits in the UK (Wytham woods), they discussed a threshold of 75% of the day spent incubating as being important. If a day is taken to be 24 hours then this 75% corresponds to 50% of the active day, so comparable but just below the threshold of 60% used for Haftorn (1981) in Norway. Following on from this pervious work, in this study we chose a definition of full incubation of 50% of the active day spent incubating to be comparable with these previous studies. The cut-off of 50% also takes account of the uneven start of incubation. Gibb (1950), Kluiver (1950), Hinde (1952) and Haftorn (1981) all note that incubation during the active day begins in the afternoon and then gradually extends to morning. We observe this same pattern in every one of the nests in this study, therefore the cut off of 50% of the active day also represents the time when incubation extends beyond midday and into the morning, defining a difference between partial daytime incubation and more steady incubation.

Figure 2: Graphs demonstrating the identification of full incubation onset. Each window is a single day (00:00-23:59) labelled with the day after clutch completion and the % of the day spent incubating. The red outline of the graph box indicates the day identified as the onset of full incubation

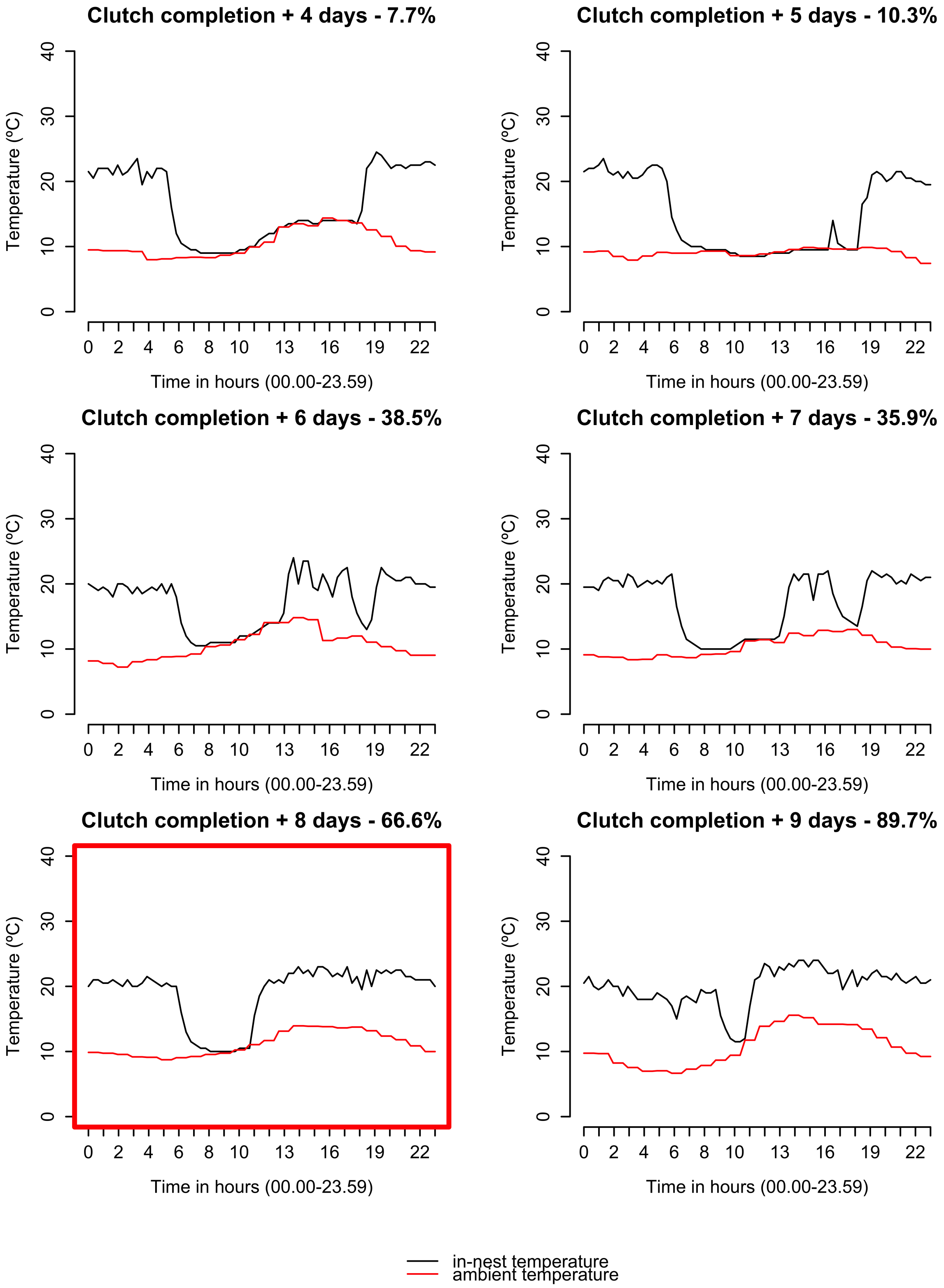

S3: Testing for potential bias in the incubation behaviour of individuals who removed iButtons

Of the 163 iButton placed in great tit nests in the 2014 breeding season, 54 were removed by females and therefore no incubation data could be obtained from these nests. It is possible that the females who rejected iButtons had a tendency towards a certain incubation behaviour, such as advancing or delaying. If this were the case, this could create a bias in the remaining sample from nests where individuals did not remove iButtons. As a result we tested whether the clutch completion to hatch interval varied significantly between nests where iButtons were removed and nests where iButtons remained for the duration of incubation. The clutch completion to hatch interval was used as a proxy for incubation behaviour as exact incubation onset was not known for nests where iButtons were removed. This is an aggregate measure that should capture both changes to the relative onset of incubation and duration but does not distinguish between the two.

An unpaired T-Test was performed to determine if the mean clutch completion to hatch interval varied between the groups and variance differences were assessed with an F-Test of variance. Both tests showed no significant differences (T = -0.57, DF = 60.3, P = 0.57 and F = 0.73, DF = 103/38, P = 0.22). The lack of a difference between the distribution of clutch completion to hatch intervals between those nests where iButtons were removed and those where they remained can also be seen visually in Figure 3.

Figure 3: Histograms of the distribution of clutch completion to hatch intervals in nests where iButtons were removed and nests where iButtons remained for the duration of incubation

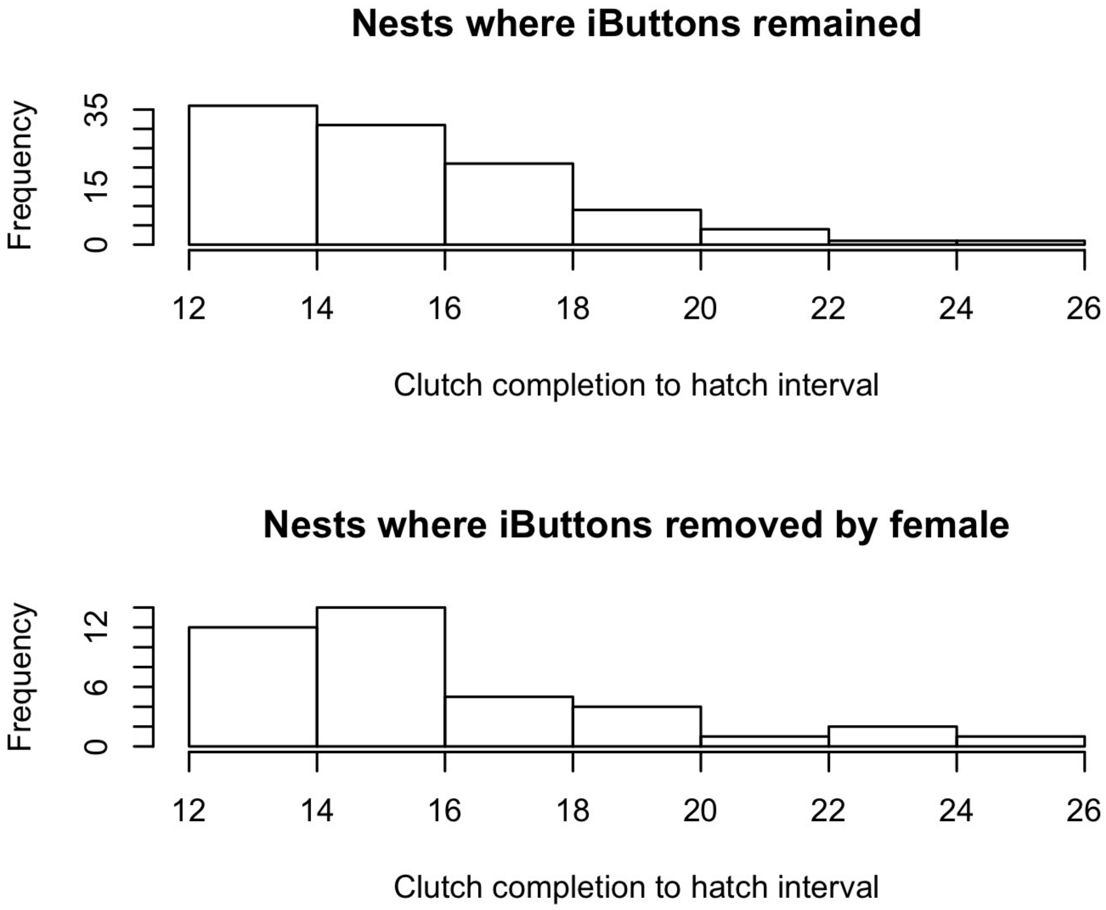

S4: Which temperature window and measure best explains variance in the relative onset of incubation?

Below we present the full results of the model selection procedure for the sliding time window analysis on the relative onset of incubation. We tested 176 candidate models with different fixed effect configurations and different temporal temperature windows and temperature measures and two null models. Shown here are the AIC results of all models with a ΔAIC of <2 (11 models) and the first model with a ΔAIC of >2. We also

present the null models, which have ΔAIC values considerably above 2.

Table 1: Results of model selection from the sliding time window analysis for the relative onset of incubation. Models with ΔAIC values of less than two are included in addition to the first model with a ΔAIC of greater than two and both null models

Model configuration	Temperature variable	AIC	Delta AIC	K
Clutch initiation * temperature	Mean - window 8	481.86	0	5
Clutch initiation * temperature	Mean - window 9	481.9	0.05	5
Clutch initiation + temperature	Mean maximum - window 8	482.86	1.01	4
Clutch initiation * temperature	Mean - window 7	482.89	1.03	5
Clutch initiation * temperature	Mean - window 10	483.26	1.4	5
Clutch initiation + temperature	Mean maximum - window 1	483.36	1.51	4
Clutch initiation + temperature	Mean maximum - window 10	483.41	1.55	4
Clutch initiation + temperature	Mean maximum - window 9	483.41	1.55	4
Clutch initiation + temperature	Mean maximum - window 7	483.69	1.83	4
temperature	Mean maximum - window 8	483.75	1.89	3
temperature	Mean maximum - window 1	483.8	1.94	3
temperature	Mean maximum - window 10	483.91	2.06	3
Clutch initiation	Null	487.47	5.62	3
Clutch initiation + clutch size	Null	489.44	7.58	4

We also present the parameter estimates for all 11 'preferred' models (ΔAIC <2). Dependent on the model configuration some table elements are missing because these variables were not included in the model itself.

Table 2: Parameter estimates for the temperature variable, clutch initiation date and their interaction for all models with ΔAIC <2. Models appear in the same order as Table 1.

Temperature measure	Temperature			Clutch initiation			Interaction		
	Est	SE	P	Est	SE	P	Est	SE	P
Mean - window 8	-5.03	1.72	0.004	-3.25	1.35	0.02	0.30	0.13	0.02
Mean - window 9	-5.40	1.83	0.004	-3.53	1.43	0.02	0.33	0.14	0.02
Mean max- window 8	-1.01	0.39	0.01	-0.09	0.06	0.10	NA	NA	NA
Mean - window 7	-4.51	1.68	0.01	-2.78	1.30	0.04	0.26	0.12	0.04
Mean - window 10	-4.94	1.84	0.01	-3.13	1.43	0.03	0.30	0.14	0.03
Mean max - window 1	-0.93	0.37	0.02	-0.09	0.06	0.13	NA	NA	NA
Mean max - window 10	-0.97	0.39	0.02	-0.09	0.06	0.12	NA	NA	NA
Mean max - window 9	-0.97	0.40	0.02	-0.09	0.06	0.11	NA	NA	NA
Mean max - window 7	-0.93	0.39	0.02	-0.09	0.06	0.12	NA	NA	NA
Mean max - window 8	-0.73	0.36	0.04	NA	NA	NA	NA	NA	NA
Mean max - window 1	-0.71	0.35	0.05	NA	NA	NA	NA	NA	NA

Results of this sliding window analysis showed two outlier values where mean temperatures for window 8 (the temperature variable of the lowest AIC model) were below 8.5 °C . To ascertain the effect of these outliers on our parameter estimates, we removed them and re-ran the model with the lowest AIC. The parameter values produced were largely the same as the model with all data, therefore, we opted to retain the maximum amount of data and present the full model in the main article. Parameter values shown in Table 3.

Table 3: Parameter values for the model with the lowest AIC with outliers removed.

Mean - window 8			Clutch initiation			Interaction		
Est	SE	P	Est	SE	P	Est	SE	P
-5.80	1.79	0.001	-3.55	1.39	0.01	0.33	0.13	0.01

S5: Which temperature window and measure best explains variance in the duration of incubation?

Here we present the full results of the model selection procedure for the sliding time window analysis on incubation duration. We tested 180 candidate models with different fixed effect configurations and different temporal temperature windows and temperature measures and two null models. 67 models had a ΔAIC of <2, including one of the null models (with clutch initiation date only). None of these models showed a significant relationship between temperature and incubation duration. Consequently here we present only the three models with the lowest AIC values (the 3rd of which is a null model) and the other null model.

Table 4: Results of model selection from the sliding time window analysis for incubation duration. Models with the three lowest ΔAIC values and the remaining null model are shown. All models also included a fixed effect of relative incubation onset

Model configuration	Temperature variable	AIC	Delta AIC	K
Temperature	Range - window 11	347.98	0	5
Temperature	Range - window 9	348.03	0.05	5
Clutch initiation	Null	348.08	0.1	4
Clutch initiation + clutch size	Null	351.03	3.05	5

Table 5: Parameter estimates for linear model of incubation duration with the lowest AIC

Range - window 11			Relative incubation onset		
Est	SE	P	Est	SE	P
0.26	0.15	0.08	-0.41	0.05	<0.001

S6: Which temperature window and measure best explains variance in the daily intensity of incubation effort?

Here we present the full results of the model selection for the temperature cues driving variation in the intensity of incubation effort. We tested 20 candidate models which included the effects of various temperature measures, day through incubation, clutch size and clutch initiation date and a null model. Models with a ΔAIC of <2 are presented below, in addition to the null model.

Table 6: Results of model selection for GLMMs for daily incubation intensity. Models with ΔAIC values of less than two are included in addition to the first model with a ΔAIC of greater than two and the null model

Model configuration	Temperature variable	AIC	Delta AIC	K
Incubation day * temperature	Daily maximum	7538.66	0	5
Incubation day + temperature	Daily maximum	7570.38	31.72	4
Incubation day + temperature	Temperature range	7600.07	61.41	4
Incubation day	Null	8249.51	710.85	3

Table 7: Parameter estimates for binomial GLMM model of proportion of the active day incubated as a function of temperature and incubation day. Random effect of individual had 0 variance

Variable	Estimate	SE	P
Daily max temperature	0.1	0.01	<0.001
Incubation day	0.22	0.02	<0.001
Daily max temperature:Incubation day	-0.008	0.001	<0.001

References

W Cresswell and R McCleery. How great tits maintain synchronization of their hatch date with food supply in response to long-term variability in temperature. *Journal of Animal Ecology*, 72:356–366, 2003.

J Gibb. The breeding biology of the great and blue titmice. *Ibis*, 92:507–539, 1950.

S Haftorn. Incubation during the Egg-Laying Period in Relation to Clutch-Size and Other Aspects of Reproduction in the Great Tit Parus major. *Ornis Scandinavica*, 12 (3):169–185, 1981.

R A Hinde. The behaviour of the great tit (parus major) and some other related species. *Behaviour supplement*, II:1–201, 1952.

H N Kluiver. Daily routines of the great tit, parus m. major l. *Ardea*, 38:99–135, 1950.
